# Functional β-Adrenoceptors Are Important for Early Muscle Regeneration in Mice through Effects on Myoblast Proliferation and Differentiation

**DOI:** 10.1371/journal.pone.0101379

**Published:** 2014-07-07

**Authors:** Jarrod E. Church, Jennifer Trieu, Radhika Sheorey, Annabel Y. -M. Chee, Timur Naim, Dale M. Baum, James G. Ryall, Paul Gregorevic, Gordon S. Lynch

**Affiliations:** 1 Basic and Clinical Myology Laboratory, Department of Physiology, The University of Melbourne, Victoria, Australia; 2 Laboratory for Muscle Research & Therapeutics Development, Baker IDI Heart and Diabetes Institute, Victoria, Australia; West Virginia University School of Medicine, United States of America

## Abstract

Muscles can be injured in different ways and the trauma and subsequent loss of function and physical capacity can impact significantly on the lives of patients through physical impairments and compromised quality of life. The relative success of muscle repair after injury will largely determine the extent of functional recovery. Unfortunately, regenerative processes are often slow and incomplete, and so developing novel strategies to enhance muscle regeneration is important. While the capacity to enhance muscle repair by stimulating β_2_-adrenoceptors (β-ARs) using β_2_-AR agonists (β_2_-agonists) has been demonstrated previously, the exact role β-ARs play in regulating the regenerative process remains unclear. To investigate β-AR-mediated signaling in muscle regeneration after myotoxic damage, we examined the regenerative capacity of tibialis anterior and extensor digitorum longus muscles from mice lacking either β_1_-AR (β_1_-KO) and/or β_2_-ARs (β_2_-KO), testing the hypothesis that muscles from mice lacking the β_2_-AR would exhibit impaired functional regeneration after damage compared with muscles from β_1_-KO or β_1_/β_2_-AR null (β_1_/β_2_-KO) KO mice. At 7 days post-injury, regenerating muscles from β_1_/β_2_-KO mice produced less force than those of controls but muscles from β_1_-KO or β_2_-KO mice did not exhibit any delay in functional restoration. Compared with controls, β_1_/β_2_-KO mice exhibited an enhanced inflammatory response to injury, which delayed early muscle regeneration, but an enhanced myoblast proliferation later during regeneration ensured a similar functional recovery (to controls) by 14 days post-injury. This apparent redundancy in the β-AR signaling pathway was unexpected and may have important implications for manipulating β-AR signaling to improve the rate, extent and efficacy of muscle regeneration to enhance functional recovery after injury.

## Introduction

Skeletal muscle is susceptible to damage associated with trauma, metabolic deficits, heritable and acquired diseases, and mechanical strains generated during contractions [1]–[4]. Although skeletal muscle has an impressive ability to regenerate after injury, the repair process is often slow and incomplete, compromised by factors such as age and pre-existing health conditions [5]. To develop treatment strategies that enhance muscle regeneration after injury and thereby improve quality of life for patients, a more comprehensive understanding of the events taking place during the regeneration of skeletal muscle is required [6].

Many of the most common and important growth factors and hormones can influence the attributes of mature and regenerating skeletal muscles. For instance, we and others have shown that stimulating β-adrenoceptors (β-ARs) with β-AR agonists (β-agonists) can enhance skeletal muscle repair after injury [6]–[9]. Recent studies have also shown that G_αi2_, a component of the β-AR signalling pathway, plays an important role in regeneration after injury and general maintenance of muscle mass [10]–[12]. However, while the capacity to enhance muscle repair via exogenous stimulation of β-ARs is now recognised, it remains unclear as to the role β-ARs play in regulating the regenerative process. To elucidate the significance of β-AR-mediated signalling in regeneration, we examined the regenerative capacity of limb muscles from mice lacking either β_1_-AR and/or the β_2_-AR. On the basis that exogenous administration of β_2_-agonists can promote regeneration, it was hypothesised that muscles from mice lacking the β_2_-AR (β_2_-KO) would exhibit impaired functional regeneration after damage compared with muscles from β_1_-KO or β_1_/β_2_-KO mice.

## Materials and Methods

### Animals

All procedures were approved by the Animal Ethics Committee of The University of Melbourne and conformed to the Australian code of practice for the care and use of animals for scientific purposes as stipulated by the National Health and Medical Research Council of Australia. Mice lacking both the β_1_- and β_2_-AR (β_1_/β_2_-KO) were purchased from The Jackson Laboratory (Adrb1_tm1Bkk_Adrb2_tm1Bkk/j_, stock #003810) and maintained by mating double homozygote KO mice. Control mice were from a C57BL/6 background, as employed previously for comparison with β_1_/β_2_-KO [13], [14] and obtained from the Animal Resources Centre (Canning Vale, WA, Australia). Mice lacking either the β_1_-AR or the β_2_-AR were purchased from The Jackson Laboratory and back crossed with C57BL/6 mice to create a heterozygous F1 generation. The heterozygous offspring of the respective strains were subsequently inbred to produce the respective β_1_-KO and β_2_-KO knockout animals and littermate controls (β_1_-WT and β_2_-WT, respectively). Mice were housed in the Biological Research Facility at The University of Melbourne under a 12 h light-dark cycle, with drinking water and standard chow provided *ad libitum*. Male mice aged 8–9 weeks were used in this study.

### Myotoxic injury

Injury was induced in the tibialis anterior (TA) or extensor digitorum (EDL) muscles of mice as described previously [15], [16]. For TA injuries, mice were anesthetized using a mixture of ketamine (76 mg/kg, i.p.) and xylazine (10 mg/kg, i.p.) and a small portion of the TA muscle of the right hindlimb was surgically exposed by a single incision through the skin. The muscle was filled to its maximal holding capacity (∼40 µl) via a single intramuscular injection with Notexin (1 µg/ml, Latoxan) using a 29-gauge needle. The wound was closed with Michel clips (Aesculap, Tuttlingen, Germany) and the mice were allowed to recover from the myotoxic injury for 7, 10 or 14 days before assessments of muscle structure and function were performed. Uninjured mice were used as controls for all experiments using the TA muscle. For EDL injuries, mice were anesthetized as described and the EDL muscle of the right hindlimb was surgically exposed by a single incision through the skin and overlying muscles. With the aid of a dissecting microscope, the EDL was filled to its maximal holding capacity (15–20 µl) via intramuscular injection at several sites with Notexin (1 µg/ml, Latoxan) using a 30-gauge needle. The wound was closed with Michel clips (Aesculap, Tuttlingen, Germany) and the mice were allowed to recover from the myotoxic injury for 2, 5, 7, 14 or 21 days. The corresponding muscle from the left (contralateral) limb served as the non-injured control muscle.

### Assessment of muscle function

Mice were anesthetized with sodium pentobarbital (60 mg/kg, i.p.) and muscle function was assessed either *in vitro* (for EDL) or *in situ* (for TA) as described previously [17], [18]. For assessment of TA muscle force producing capacity *in situ*, the muscles of anesthetised mice were stimulated by supramaximal 0.2 ms square wave pulses of 350 ms duration, delivered via two wire electrodes placed adjacent to the deep peroneal branch of the sciatic nerve. Force generated was measured via attachment of a force transducer to the distal tendon of the muscle. For assessments of EDL muscle function *in vitro* the muscles from each limb were surgically excised from anesthetized mice and transferred to a custom-built organ bath filled with Krebs-Ringer solution (in mM: NaCl, 1.37; NaHCO_3_, 24; D-glucose, 11; KCl, 5; CaCl_2_, 2; NaH_2_PO_4_, 1; MgSO_4_, 0.487, pH 7.4) supplemented with d-tubocurarine chloride (0.293 mM), bubbled with Carbogen (5% CO_2_ in O_2_) and maintained at 25°C, where the proximal tendon was attached to a micro-manipulator (to facilitate adjustment of muscle length to optimal), and the distal tendon was attached to a force transducer. The EDL was stimulated by supramaximal (40 V) 0.2 ms square wave pulses of 350 ms duration delivered via platinum plate electrodes that flanked the full length of each muscle.

For both *in situ* and *in vitro* assessments, optimal muscle length for contraction (L_o_) was defined as the muscle length at which maximal isometric twitch force (P_t_) was attained and maximal isometric tetanic force (P_o_) was determined from the plateau of the frequency-force relationship. P_o_ was normalized to the cross-sectional area of the muscle (CSA; calculated using L_o_ and muscle mass) in order to determine maximum specific isometric tetanic force (sP_o_), a measurement that allows for relative comparisons of force production between muscles of different sizes.

Immediately following the functional assessments, muscles were carefully trimmed of any adherent non-muscle tissue and tendons, and weighed on an analytical balance. Muscles were then mounted in Tissue-Tek OCT embedding medium, frozen rapidly in thawing isopentane, and stored at -80°C for later histological and biochemical analyses. The mice were killed by surgical excision of the heart while still anesthetized deeply.

### Muscle morphology

Serial transverse cryosections (5 µm) were cut from the midbelly of each muscle and placed onto glass slides (Superfrost Plus, Menzel-Gläser, Kensington, VIC, Australia). General muscle histology was determined by staining sections with hematoxylin and eosin (H & E) to visualize muscle fibers, and digital images of stained sections were obtained using a microscope equipped with digital camera (Carl Zeiss, Wrek, Göttingen, Germany) supported by associated imaging software (Axiovision V4.7.1.0).

### Quantitative RT-PCR

Muscle samples were homogenized individually and mRNA extracted using an RNEasy fibrous tissue RNA extraction kit (Qiagen), according to manufacturer's instructions. The concentration and quality of RNA in each sample was determined using a Nanodrop 2000 (Thermo Scientific) and the extracted mRNA was stored at −80°C. mRNA was transcribed into cDNA using the Superscript VILO cDNA synthesis kit (Invitrogen) according to manufacturer's instructions, and stored at −20°C until use. Quantitative RT-PCR was performed using an iCycler Thermal Cycler (Bio-Rad) with SYBR Green supermix (Quantace). Primers were designed using the Perfect Primer online program (Invitrogen) and are listed in Table 1. Due to the severity of the myotoxic damage caused by Notexin at the earlier time points [15], we chose not to use the ΔCt method of analysis as we could not be certain that any reference gene used would remain unchanged between treatment groups. We instead opted to measure the cDNA concentration of each sample using the Quant-iT OliGreen ssDNA Assay Kit (Molecular Probes) and to normalize our data to cDNA content as described previously [19], [20].

**Table 1:** PCR primer sequences.

	Genbank accession number		Primer Sequences (5′-3′)
**Myf5**	NM_008656	**F**	5′- AACCAGAGACTCCCCAAGGT-3′
		**R**	5′- AGCTGGACACGGAGCTTTTA-3′
**MyoD**	NM_010866	**F**	5′- AGTGAATGAGGCCTTCGAGA-3′
		**R**	5′- GCATCTGAGTCGCCACTGTA-3′
**Myogenin**	NM_031189	**F**	5′- CACTCCCTTACGTCCATCGT-3′
		**R**	5′- CAGGACAGCCCCACTTAAAA-3′
**MRF4**	NM_008657	**F**	5′- GGCTGGATCAGCAAGAGAAG-3′
		**R**	5′- AAGAAAGGCGCTGAAGACTG-3′
**TNF-α**	NM_013693	**F**	5′- GGCCTTCCTACCTTCAGACC-3′
		**R**	5′- AGCAAAAGAGGAGGCAACAA-3′
**IL-6**	NM_031168	**F**	5′- CCGAGAGGAGACTTCACAG-3′
		**R**	5′- TCCACGATTTCCCAGAGAACC-3′
**CD68**	NM_009853	**F**	5′- TCCAAGCCCAAATTCAAATC-3′
		**R**	5′- ATTGTATTCCACCGCCATGT-3′
**F4/80**	NM_010130	**F**	5′- CATCAGCCATGTGGGTACAG-3′
		**R**	5′- CATCACTGCCTCCACTAGCA-3′

### Cell culture

Primary mouse myoblasts were isolated using a protocol adapted from Huang and colleagues [21]. Briefly, mice were anesthetized and killed by cardiac excision and the quadriceps, TA and gastrocnemius muscles were excised, cleaned of all connective tissue, and rinsed in ice-cold culture media (Ham's Nutrient Mix F-12). The muscles were placed in a digest solution consisting of culture media supplemented with 1% Type II collagenase and 0.5% Dispase (Invitrogen), minced with a clean pair of scissors, and incubated in a shaking incubator at 37°C for 90 min. The suspension was filtered through a 100 µm cell filter (BD Biosciences), pelleted (1500×g, 5 min) and resuspended in complete growth media (Ham's Nutrient Mix F-12 supplemented with 20% FBS, 1% L-glutamine, 1% antibiotic-antimycotic and 5 ng/ml rhFGF). The cell suspension was plated into uncoated cell culture dishes overnight to allow fibroblasts to adhere, after which the non-adherent cells were aspirated and plated into a fresh uncoated dish for a further 24 hrs to remove as many fibroblasts as possible. Following the second overnight incubation, non-adherent cells were aspirated and seeded into a cell culture dish pre-coated with extracellular matrix (ECM; Sigma). Cells were expanded in culture for approximately 4 weeks, after which myoblast proliferation and differentiation were assessed.

For proliferation assays, isolated primary myoblasts were seeded into ECM-coated 96 well plates at a density of 1×10^4^ cells/well and incubated in complete growth media for 72 hrs. Subsequently, the cells were fixed (3.7% formaldehyde, 10 min, 25°C), rinsed twice with PBS, and incubated with DAPI (0.235 µM diluted in PBS, 15 min, 25°C). The number of cell nuclei was quantified using an inverted fluorescence microscope and camera (Axiovert 40, Carl Zeiss) and associated imaging software (Axiovision V4.7.1.0, Carl Zeiss).

For differentiation assays, the isolated primary myoblasts were seeded into ECM-coated 12-well plates at a density of 5×10^5^ cells/well. After incubating for 2 hr to permit cell attachment, growth media was replaced with differentiation media (DMEM supplemented with 2% horse serum and 1% L-glutamine). Cells were cultured in differentiation-inducing conditions for 3 days, with differentiation media replaced daily. To examine attributes of differentiation, cells were imaged with an inverted microscope and camera (Axiovision 40, Carl Zeiss) controlled by associated software (Axiovision V4.7.1.0). Differentiation was measured as total area (µm^2^) in the field of view occupied by multinucleated myotubes.

### Statistical analyses

All values are reported as mean ±SEM. Groups were compared using an unpaired Student's *t*-test, or a one-way or two-way ANOVA and Bonferroni's post hoc multiple comparison procedure where appropriate. Myofiber cross-sectional area (CSA) is not normally distributed, and so medians were compared using a non-parametric Mann-Whitney test by ranks. All statistical analyses were performed using Prism version 3 software (GraphPad Software, Inc. La Jolla, CA). In all cases significance was defined as P<0.05.

## Results

### Deletion of specific β-ARs has differential effects on muscle mass and force producing capacity

**Figure 1:**
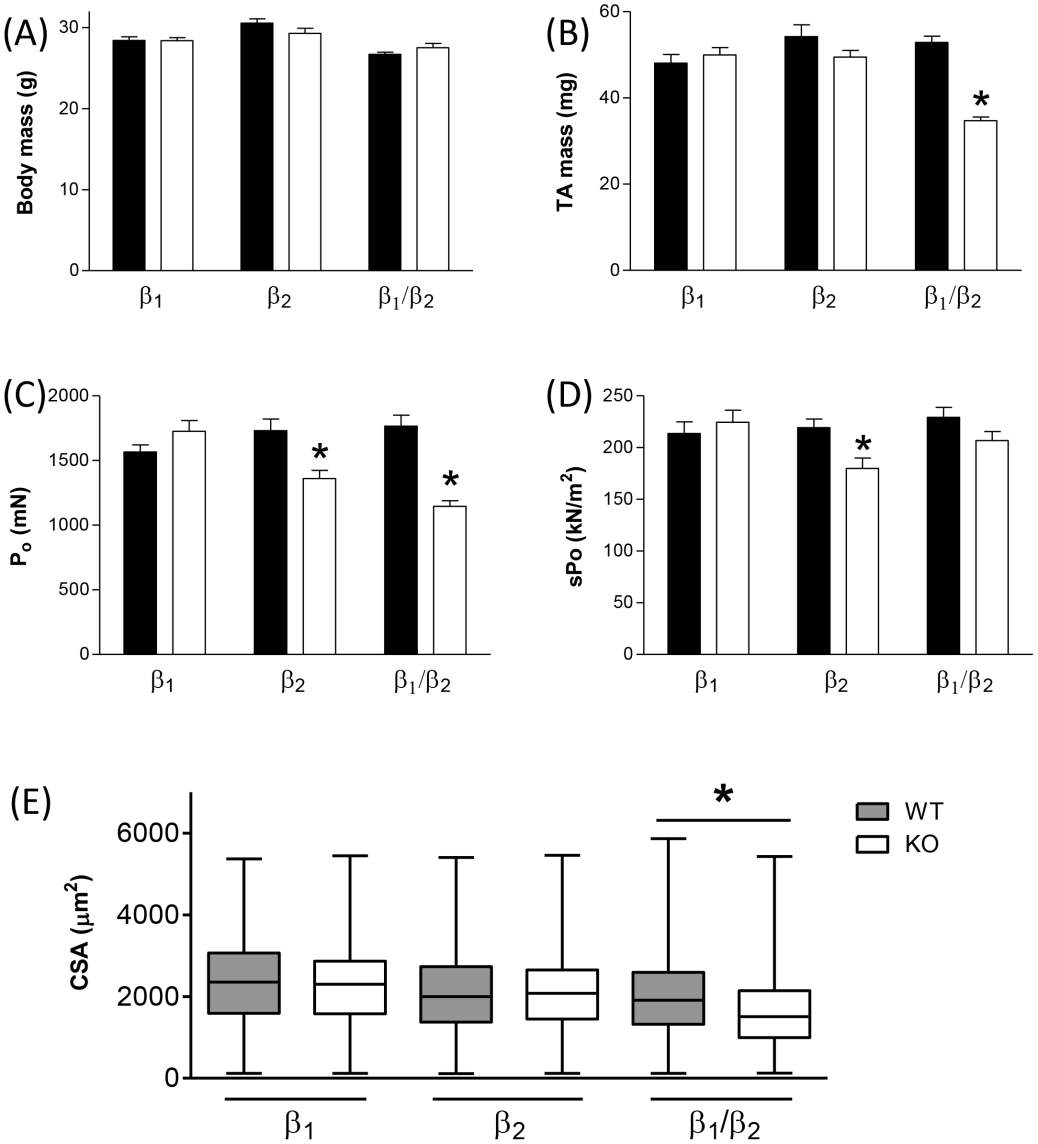
Basic morphology and function of the TA muscles of different β-KO mouse strains. (A) Body masses for the different β-KO mouse strains (white bars) and their respective controls (black bars). No significant differences were observed across the strains. (B) Comparison of TA muscle mass in β-KO mouse strains (white bars) and their respective controls (black bars). There was no significant difference in TA mass for either β_1_-KO or β_2_-KO mice when compared with littermate controls, but β_1_/β_2_-KO mice had a significantly lower TA mass than control (*P<0.05 compared with control, *t*-test, *n* = 5-6 per group). (C) and (D) maximal tetanic force production by TA muscles measured as either absolute force (P_o_) or force normalized to muscle size (specific force, or sP_o_). β_1_-KO mice had no significant differences in maximal force whether expressed as either P_o_ or sP_o_. In contrast, β_2_-KO mice had significant deficits in maximal force when expressed as either P_o_ or sP_o_. β_1_/β_2_-KO mice had significantly lower maximal forces than control when expressed as P_o_, but there was no significant deficit in force production when force was normalized to muscle size (sP_o_) (*P<0.05 compared with control, *t*-test, *n* = 5–6 per group). (E) Comparison of cross-sectional area (CSA) of TA muscle fibers from β-KO mouse strains (white bars) and their respective controls (grey bars). Neither β_1_-KO nor β_2_-KO mice had significant changes in muscle fiber CSA compared with their wild-type littermates, but fiber CSA was significantly lower in TA muscles from β_1_/β_2_-KO mice compared with control. Data for each strain were pooled from >1500 fibers from *n*≥9 mice (*P<0.05, Mann-Whitney test by ranks).

We first compared the morphology and functional characteristics of TA muscles from the different β-AR KO strains of mice. We found no significant differences in body mass between any of the β-KO mice and their respective controls (Fig. 1A). When we compared the muscle mass between the strains, neither β_1_-KO mice nor β_2_-KO mice were different from their respective controls, but TA muscles of β_1_/β_2_-KO mice were significantly smaller (Fig. 1B). When maximal force was expressed as absolute force (P_o_), β_1_-KO mice did not exhibit differences in force production compared with controls, but both β_2_-KO mice and β_1_/β_2_-KO mice produced less force than their controls (Fig. 1C). When maximal force production was normalized to muscle size (sP_o_), β_1_-KO mice still exhibited no differences in force production and β_2_-KO mice still exhibited deficits in force production, but β_1_/β_2_-KO mice demonstrated equivalent functional capacity compared with control (Fig. 1D). Examining muscle fiber cross-sectional area (CSA) for the various β-KO mouse strains revealed that, as for TA muscle mass, neither the muscles of β_1_-KO mice nor β_2_-KO mice were different from the muscles of their respective littermate controls, but that muscle fiber CSA in β_1_/β_2_-KO mice was significantly smaller than muscle fibres from wild type mice (Fig. 1E). We also analyzed a number of twitch characteristics of TA muscles from the β-KO mice (Table 2) and found that the muscles of β_1_-KO mice did not differ from control mice, but the muscles of both β_2_-KO mice and β_1_/β_2_-KO mice elicited reduced peak twitch force (P_t_) and a reduced rate of contraction (dP_t_/dt) compared with the muscles of controls. An analysis of the frequency-force relationship found no significant differences between β_1_-KO mice and control, but significant differences between β_2_-KO mice and control, and β_1_/β_2_-KO mice and control (Figure S2).

**Table 2:** Selected isometric twitch contractile properties of uninjured TA muscles from β-KO mice.

	β_1_-WT	β_1_-KO	β_2_-WT	β_2_-KO	C57BL/6	β_1_/β_2_-KO
*n*	5	5	5	5	6	5
**P_t_ (mN)**	472±20	502±38	513.8±30.9	425.6±21.6*	457±17	369±27*
**TPT (ms)**	17.3±0.8	16.7±0.8	15.5±0.5	16.6±1.3	14.4±0.6	15.6±0.4
**½ RT (ms)**	17.4±2.0	16.4±1.1	14.5±1.1	13.8±1.9	12.2±1.0	16.1±1.6
**dP_t_/dt (mN/ms)**	67.8±2.8	70.4±1.7	79.7±2.9	67.13±3.7*	73.8±1.4	63.2±4.7*

P_t_ - peak twitch tension; TPT - time to peak twitch tension; ½ RT - one-half relaxation time; dP_t_/dt - maximum rate of force development during a twitch contraction.

*P<0.05 vs. vehicle control, Student's unpaired *t*-test.

### Deletion of β_2_-ARs impairs the functional performance of regenerating muscles

**Figure 2:**
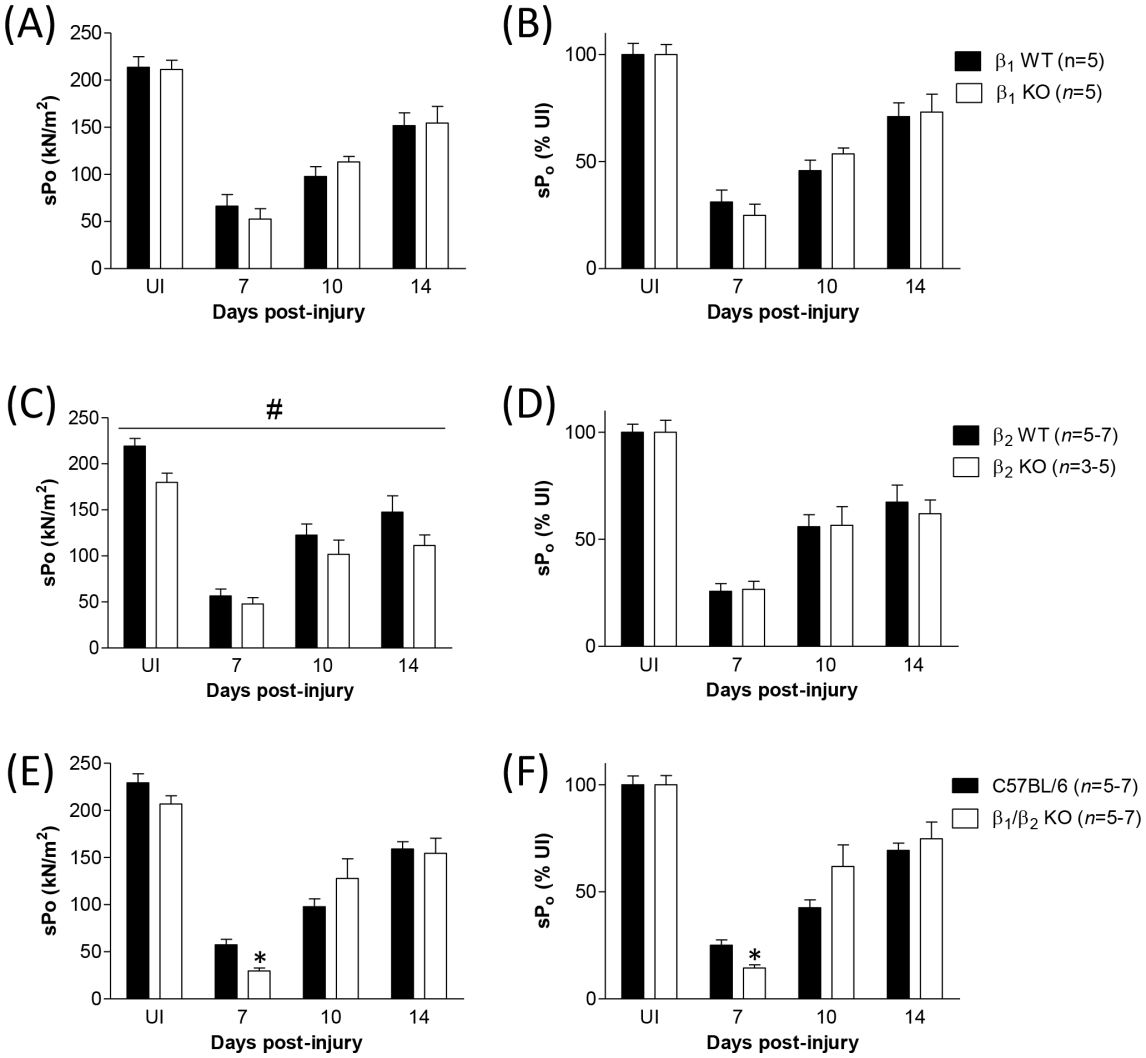
Maximal tetanic force production by regenerating TA muscles of different strains of β-KO mice after injury and measured as either specific force (sP_o_; a, c, e) or specific force normalized to uninjured muscles (%UI; b, d, f). β_1_-KO mice did not exhibit significant deficits in maximal force during regeneration (A and B). When maximal force was expressed as sP_o_, β_2_-KO mice had force deficits during all stages of regeneration (C; #*P*<0.05, strain main effect, 2-way ANOVA), although a Bonferroni post-hoc test did not detect any differences at specific time points. When force production was normalised to uninjured muscles (%UI), there was no difference between β_2_-KO mice and controls during regeneration (D). β_1_/β_2_-KO mice did not exhibit delayed regeneration over the 14 day period when analysed with a 2-way ANOVA, but there was a significant deficit in maximal force by β_1_/β_2_-KO mice (expressed as either sP_o_ or %UI) at 7 days post-injury when the time point was analysed in isolation (*P<0.05 compared with control, *t*-test, *n* = 7) (E and F).

We next examined the recovery of force production in the TA muscles of the various β-KO mice at 7, 10 and 14 days after myotoxic damage. When force production was expressed as absolute force (P_o_), we found that β_1_-KO mice displayed no differences in maximal force production during regeneration, whereas both β_2_-KO mice and β_1_/β_2_-KO mice produced significantly less force compared with controls (Figure S1). When force was normalized to muscle size (sP_o_) maximal force production was unchanged in β_1_-KO mice from controls (Fig. 2A). Maximal force in β_2_-KO mice was significantly lower than controls throughout regeneration (Fig. 2C), but force production by β_1_/β_2_-KO mice was no longer significantly different from control over the 14 days of regeneration (Fig. 2E). In order to take into account the lower sP_o_ of uninjured muscles from β_2_-KO mice, we then expressed force production during regeneration as a percentage of that produced by uninjured muscles (%UI). When maximal force was expressed this way, we found that none of the β-KO strains exhibited any deficits in the restoration of force producing capacity compared with controls (Fig. 2B, 2D and 2F). When the data at 7 days post-injury were analyzed separately in order to assess the earliest time point in isolation, β_1_/β_2_-KO mice had impaired force production compared with controls whether expressed as P_o_, sP_o_ or %UI.

**Figure 3:**
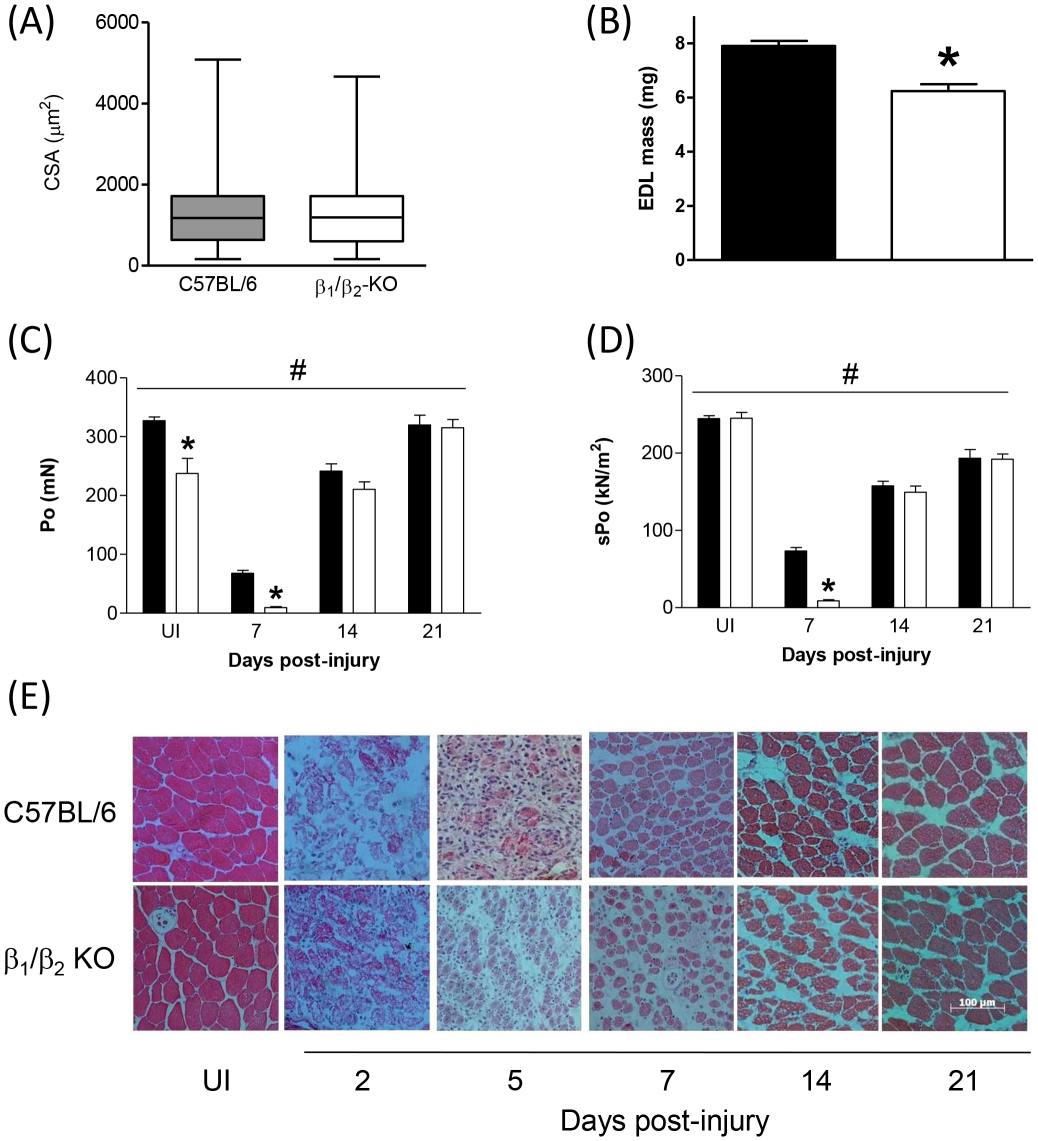
Morphological properties and functional regeneration after myotoxic damage in the EDL muscles of β_1_/β_2_-KO mice and C57BL/6 controls. (A) Comparison of muscle fiber CSA in uninjured EDL muscles. There was no significant difference in muscle fiber CSA between β_1_/β_2_-KO mice (white bar) when compared with C57BL/6 (black bar). Data were pooled from >1500 fibers from *n* = 5 mice (B). Comparison of EDL muscle mass. β_1_/β_2_-KO mice had lower mass than C57BL/6 controls (*P<0.05 compared with control, *t*-test, *n* = 29 and 21, respectively). (C) and (D) Maximal force by regenerating EDL muscles expressed as either absolute force (P_o_) or force normalized to muscle size (specific force, or sP_o_). β_1_/β_2_-KO mice (white bars) had lower maximal forces when expressed as either P_o_ or sP_o_ (^#^ *P*<0.05, strain main effect, 2-way ANOVA), with significant deficits at 7 days post-injury (*P<0.05, Bonferroni's post hoc multiple comparison procedure). (E) H&E stained sections of fibres from regenerating EDL muscles of C57BL/6 or β_1_/β_2_-AR KO mice. Note the qualitative differences in regeneration at 7 and 14 days post-injury.

In order to more comprehensively assess the role of β-ARs in skeletal muscle we also compared morphological characteristics of EDL muscles between β_1_/β_2_-KO and C57BL/6 control mice. Our findings for the EDL muscles closely mirrored those for the TA muscle; viz., muscle fiber CSA was not significantly different between β_1_/β_2_-KO mice and C57BL/6 controls (Fig. 3A) and uninjured muscles from β_1_/β_2_-KO mice were significantly smaller than controls (Fig. 3B) and produced lower maximal absolute forces (Fig. 3c and Table 3). Similarly, when maximal force was normalized to muscle cross-sectional area (sP_o_) there were no significant differences between β_1_/β_2_-KO mice and controls (Fig. 3D and Table 3). When twitch characteristics of uninjured EDL muscles were compared between the two strains, we found that as for the TA muscles, both twitch force (P_t_) and rate of contraction (dP_t_/dt) were significantly lower in the β_1_/β_2_-KO mice (Table 3). Following myotoxic damage, EDL muscles from β_1_/β_2_-KO mice exhibited impaired restoration of force production over 21 days, with a particularly significant deficit at 7 days post-injury (Fig. 3C and 3D). In addition to the delayed restoration of contractile force in recovery EDL muscles, qualitative differences in muscle architecture were observed at both 7 and 14 days post-injury (Fig. 3E).

**Table 3:** Selected isometric twitch contractile properties of uninjured EDL muscles from β_1_/β_2_-KO mice and C57BL/6 controls.

	C57BL/6	β_1_/β_2_-KO
*n*	26	23
**Po**	327.1±6.5	237.4±26.0*
**sPo**	244.6±3.9	245.0±7.5
**P_t_ (mN)**	83.2±3.1	65.4±3.4*
**TPT (ms)**	20.9±0.4	21.5±0.5
**½ RT (ms)**	26.4±0.7	27.9±1.1
**dP_t_/dt (mN/ms)**	13.4±0.6	11.3±0.5*

P_t_ - peak twitch tension; TPT - time to peak twitch tension; ½ RT - one-half relaxation time; dP_t_/dt - maximum rate of force development during a twitch contraction.

*P<0.05 vs. vehicle control, Student's unpaired *t*-test.

### Expression of inflammatory markers and myogenic regulatory factors during early regeneration is potentiated in muscles lacking β_1_- and β_2_-ARs

**Figure 4:**
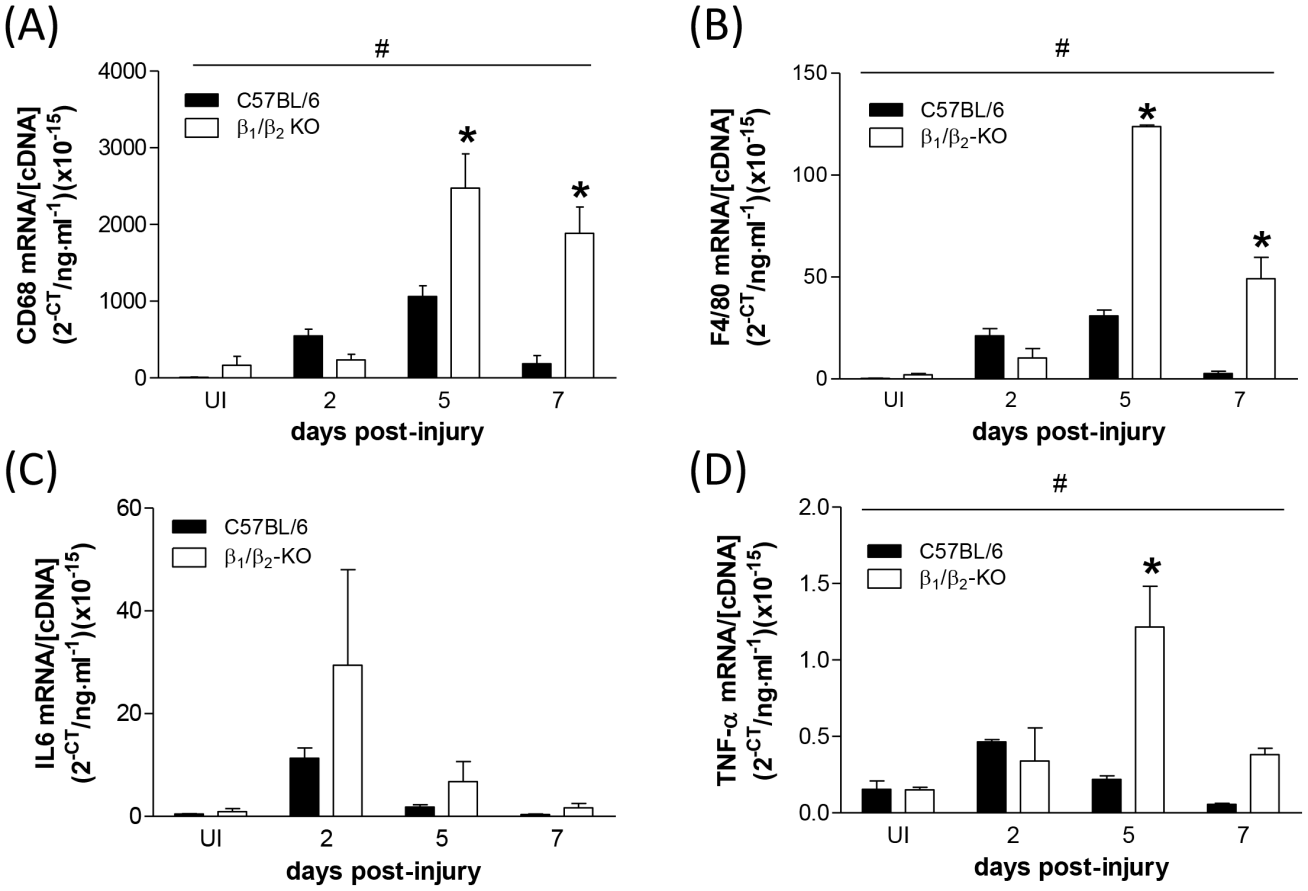
mRNA expression of (A) CD68, (B) F4/80, (C) IL-6 and (D) TNF-α in regenerating TA muscles of C57BL/6 and β_1_/β_2_-KO mice. β_1_/β_2_-KO mice had higher expression of all four markers of inflammation and macrophage infiltration during regeneration (*n* = 3; #*P*<0.05, strain main effect, 2-way ANOVA; *P<0.05, Bonferroni's post hoc multiple comparison procedure).

As we had detected impaired force production in β_1_/β_2_-KO mice during the early stages of regeneration (at 7 days post-injury), we examined the expression of both inflammatory markers and myogenic regulatory factors in TA muscles from β_1_/β_2_-KO mice and C57BL/6 control mice immediately after myotoxic injury and during early regeneration. Quantitative RT-PCR for the cytokines interleukin-6 (IL-6) and tumour necrosis factor-α (TNF-α) revealed an increase in cytokine expression in C57BL/6 mice after myotoxic damage, which peaked at 2 days post-injury (Fig. 4C and 4D). Injury-induced expression of TNF-α was significantly higher in β_1_/β_2_-KO mice than in C57BL/6 controls (Fig. 4D; 2-way ANOVA, main strain effect), and expression of IL-6 showed a non-significant trend toward higher expression in β_1_/β_2_-KO mice (Fig. 4C). Expression of the macrophage markers CD68 and F4/80 revealed an increase in macrophage infiltration in injured muscles from both β_1_/β_2_-KO and C57BL/6 mice which peaked at 5 days post-injury (Fig. 4A & B). Injury-induced expression of CD68 and F4/80 was significantly higher in β_1_/β_2_-KO mice than in uninjured controls (2-way ANOVA, main strain effect).

**Figure 5:**
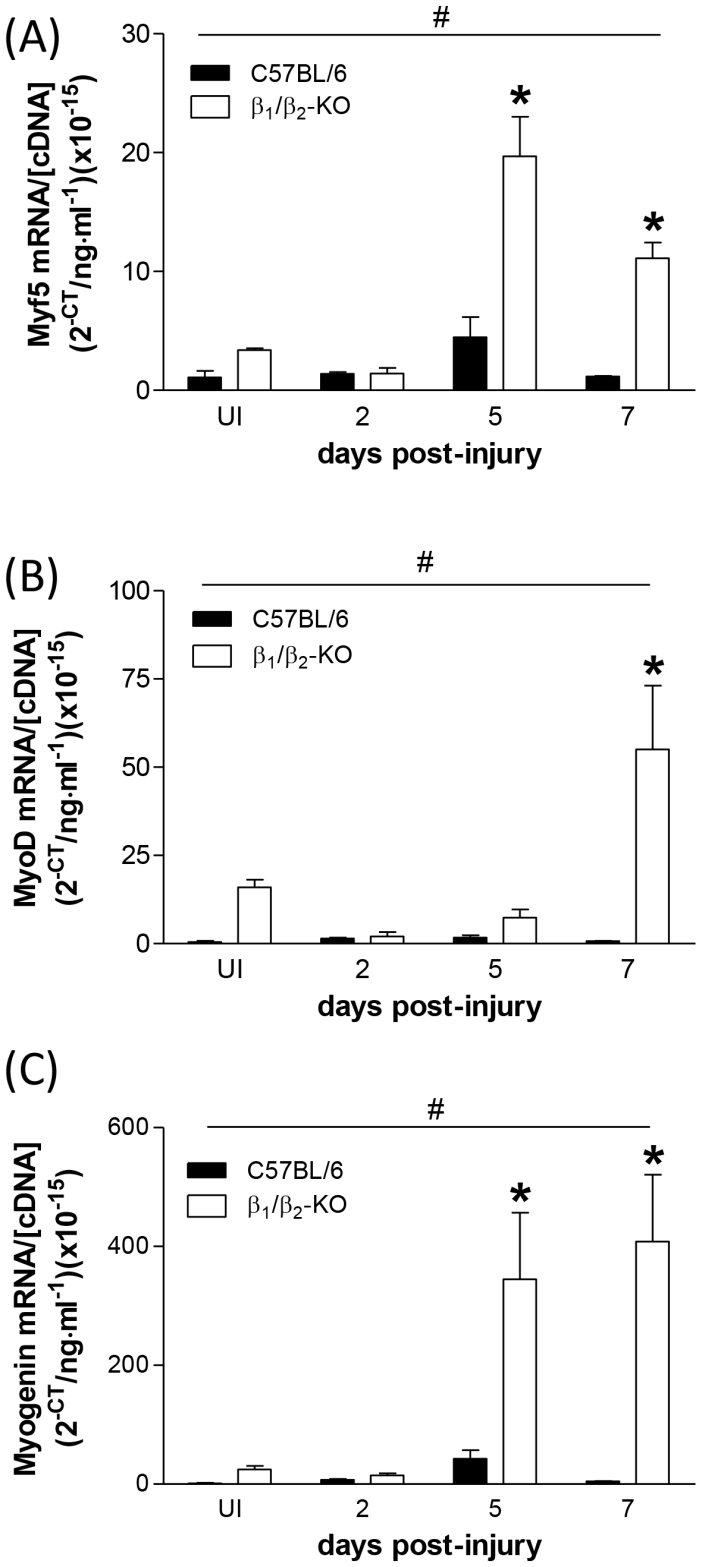
mRNA expression of (A) Myf5, (B) MyoD and (C) myogenin in regenerating TA muscles of C57BL/6 and β_1_/β_2_-KO mice. β_1_/β_2_-KO mice had higher expression of all three MRFs during regeneration (*n* = 3; #*P*<0.05, strain main effect, 2-way ANOVA; *P<0.05, Bonferroni's post hoc multiple comparison procedure).

An examination of myogenic regulatory factor (MRF) mRNA revealed that Myf5, MyoD and myogenin expression all increased after myotoxic damage, peaking at 5 days post-injury (Fig. 5A, B and C). Expression of all three MRFs was significantly higher in β_1_/β_2_-KO mice (2-way ANOVA, main strain effect), and also seemed to be more sustained than the increases in control muscles, with MyoD and myogenin in particular remaining elevated for longer than 5 days post-injury (Fig. 5A, B and 5C).

### Deletion of β_1_- and β_2_-ARs increases myoblast proliferation and inhibits differentiation ex vivo

**Figure 6:**
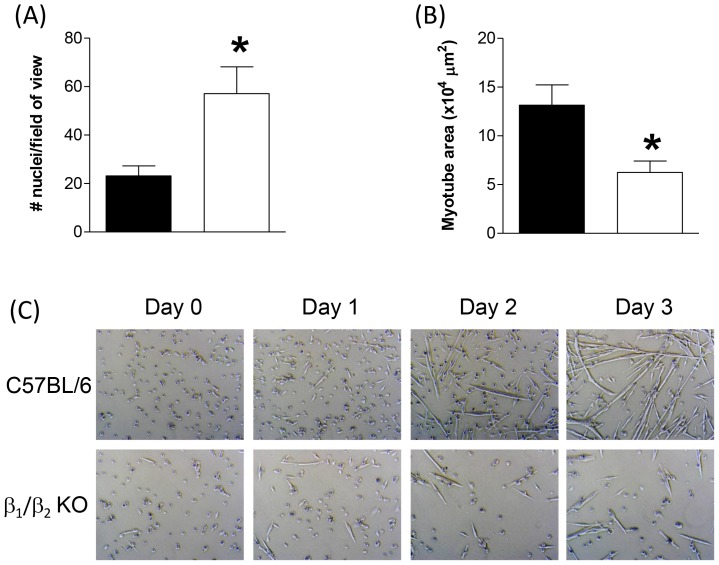
(A) Proliferation of primary myoblasts isolated from either C57BL/6 (black bars) or β_1_/β_2_-KO (white bars) mice. Myoblasts from β_1_/β_2_-KO mice proliferated more rapidly than those from C57BL/6 controls (*P<0.05 compared with control, *t*-test, *n* = 6). (B) and (C) Differentiation of primary myoblasts isolated from either C57BL/6 (black bars) or β_1_/β_2_-KO (white bars) mice. Myoblasts from β_1_/β_2_-KO mice had significantly impaired differentiation compared with those from C57BL/6 controls (*P<0.05 compared with control, *t*-test, *n* = 6).

To directly determine the proliferation and differentiation of myoblasts from β_1_/β_2_-KO mice without the confounding variables inherent to the whole-body knockout mouse, we isolated and cultured myoblasts from β_1_/β_2_-KO mice and C57BL/6 controls. We found that myoblasts from β_1_/β_2_-KO mice proliferated more rapidly and differentiated less effectively than those from C57BL/6 controls (Fig. 6).

## Discussion

Although exogenous stimulation of β-ARs has been shown to enhance mammalian muscle fiber regeneration [6]–[9], and recent studies have also implicated components of the β-AR signalling pathway in muscle regeneration and hypertrophy [10]–[12], the contribution of β-ARs to the muscle regenerative process *per se* is less well understood. The findings from the present study have addressed this gap in existing knowledge by examining the functional capacity of skeletal muscles from β_1_-, β_2_- and β_1_/β_2_-KO mice. While many previous studies have examined β-KO mice in a cardiovascular context, the attributes of skeletal muscle are less well described.[22]. We found that the mass of TA muscles from either β_1_-KO mice or β_2_-KO mice were similar to respective controls, whereas the TA muscles from β_1_/β_2_-KO mice were significantly smaller than control. Muscle fiber CSA in the various strains closely mirrored muscle mass, with neither of the single knockout strains displaying significant differences in CSA while the double knockouts had a significant reduction. Hinkle and colleagues [22] also found no difference in TA mass between β_1_-KO mice and control, but noted that both β_2_-KO mice and β_1_/β_2_-KO mice exhibited significantly reduced TA muscle mass than controls, from which they concluded that muscle size was positively regulated by β_2_-ARs but not β_1_-ARs. In the present study, we did not observe any significant difference in TA muscle mass in β_2_-KO mice, but did note a significant reduction in TA mass in β_1_/β_2_-KO mice. The reason for the discrepancy between the studies is unclear but may reflect the back-crossing onto a different background strain (C57BL/6) in the present study.

The muscles of mice lacking β_1_-ARs were identical to controls for every functional parameter measured, but muscles from both β_2_-KO mice and β_1_/β_2_-KO mice produced less absolute force than controls (whether twitch force or tetanic force) and exhibited a slower rate of force generation. The only difference we observed in functionality between the muscles of β_2_-KO mice and β_1_/β_2_-KO mice was that of specific force (sP_o_). When expressed this way muscles from β_1_/β_2_-KO mice produced similar forces to control, whereas force production by β_2_-KO mice remained significantly depressed relative to control muscles. Taken together, our data indicate that the loss of β_2_-ARs has profound effects on skeletal muscle morphology and contractile function, but the loss of β_1_-ARs does not significantly impact on either parameter. This is perhaps not surprising given that β_1_-ARs make up only about 7–10% of the total β-ARs in skeletal muscle [23]. It is worth noting however that the expression of β_1_-ARs is relatively higher in slow-twitch muscles such as the soleus, and that the present study examined the TA and EDL that are both predominantly fast-twitch muscles. We therefore cannot exclude the possibility that the loss of β_1_-ARs may have an impact on contractile function in slow twitch muscles such as the soleus. These findings also confirm previous studies demonstrating that β_2_-AR signaling has significant effects on force producing capacity [24], [25].

In the context of muscle regeneration, we found that the muscles from β_1_-KO mice did not show any significant differences in force production after myotoxic injury compared with controls. In contrast, muscles from β_2_-KO mice displayed significant deficits in force production during regeneration when measured as either absolute force or specific force. When we accounted for the fact that the muscles of β_2_-KO mice were returning to a lower absolute force (i.e. we expressed force production as ‘% uninjured’), we found no significant differences in the restoration of force production in regenerating muscles between β_2_-KO mice and controls. Similarly, muscles from β_1_/β_2_-KO (double knockout) mice exhibited significant differences in absolute force production compared with controls, but when force was normalized to either muscle cross-sectional area (sP_o_) or to force production by uninjured muscles (% uninjured) there was no difference in the restoration of force over the 14 day period of regeneration. If the data are normalized to reflect the intrinsic differences in muscle strength and the entire 14 day period of regeneration is analysed in its entirety, then a lack of β-ARs (either β_1_-ARs, β_2_-ARs or both β_1_- and β_2_-ARs) does not appear to affect the restoration of muscle function after myotoxic damage. If, however, we confine our analysis to the earliest time point of the regeneration period (i.e. 7 days post-injury), a different pattern emerges. In this case, early regeneration of the TA muscle in both β_1_-KO mice and β_2_-KO is unaffected compared with controls, whereas the muscles of β_1_/β_2_-KO mice exhibit significant force deficits. Our results suggest a level of redundancy between the two subtypes of β-AR. This finding may be confounded by these mice having lifelong knockout of β-ARs, which may promote possible compensatory adaptations in β-AR signaling that accompany such models. In addition, it seems unlikely that β_1_-ARs, which comprise only 7–10% of β-ARs within muscle [23] could compensate for the loss of β_2_-ARs. Nonetheless, the possibility of β-AR redundancy requires further investigation, possibly by other experimental models such as inducible gene knock-out, or in vivo knockdown of β-ARs with siRNA, that would be less likely to be confounded by compensatory alterations in signaling.

To confirm our findings in the TA muscle, we also examined the characteristics of the EDL muscles. Since our findings from the TA muscle had indicated only β_1_/β_2_-KO mice exhibited differences in regeneration after myotoxic injury, we confined our analysis of EDL morphology and function to β_1_/β_2_-KO mice and C57BL/6 controls. Uninjured EDL muscles from β_1_/β_2_-KO mice exhibited similar differences in morphology and function to TA muscles; i.e. decreased muscle mass, decreased twitch force and rate of contraction, and decreased absolute tetanic force when compared with C57BL/6 controls. Furthermore, EDL muscles from β_1_/β_2_-KO mice displayed similar deficits in regeneration at 7 days post-injury, confirming our findings in the TA muscle and further suggesting that the loss of both β_1_- and β_2_-ARs affects the earliest period of regeneration after myotoxic damage.

Skeletal muscle regeneration is preceded by a well-defined and highly coordinated inflammatory response, involving the infiltration of neutrophils and macrophages at the site of damage and subsequent release of pro- and anti-inflammatory cytokines [26]. Non-cytokine anti-inflammatory mediators are also able to modulate the inflammatory process, including glucocorticoids, adenosine, and endogenous β-agonists [27]. β-ARs are not only present in skeletal muscle [23], but have also been detected on the surface of inflammatory cells such as mast cells, eosinophils, neutrophils and macrophages [28]. To determine the degree of macrophage infiltration in regenerating muscles, we examined the expression of the macrophage-specific markers F4/80 and CD68 [29]. The expression of both macrophage markers was significantly increased in β_1_/β_2_-KO than in controls. Previous studies have revealed that macrophages incubated with LPS have an increased expression of TNF-α and IL-6, which is inhibited by the β-agonist clenbuterol [30], [31] and that administering the β-AR antagonist propranolol can potentiate the release of inflammatory cytokines in vivo [32], [33]. Due to the well-characterized increase in TNF-α and IL-6 in response to inflammation, as well as the evidence that their expression is controlled to some degree by β-AR signaling, we examined these two cytokines as a measure of inflammation and found that both cytokines were increased acutely after injury, with maximal expression at 2 days post-injury in control mice. This correlates well with previous studies from our laboratory where we have reported that oedema and immune cell infiltration are maximal at this time [15]. In the present study, TNF-α was significantly more elevated in β_1_/β_2_-KO mice, and IL-6 mRNA showed a non-significant trend toward higher expression than in regenerating muscles from control mice, suggesting that the acute immune response to injury in β_1_/β_2_-KO mice is exacerbated by the lack of β-ARs. Taken together, these data suggest that the inflammatory response and subsequent macrophage infiltration after injury is acutely higher in the β_1_/β_2_-KO mice compared with control.

To ascertain whether the observed force deficits in muscles from β_1_/β_2_-KO mice at 7 days post-injury were associated with impaired myofiber regeneration, we assessed the expression of the myogenic regulatory factors (MRFs) responsible for muscle formation. Previous studies have demonstrated that clenbuterol represses the expression of MyoD and myogenin in denervated rat soleus muscles [34], and that clenbuterol administration increased myogenin expression in immobilized rat plantaris muscles [35]. Clenbuterol administration also increased MyoD expression in rat soleus muscles [36]. In the present study, Myf5 (the transcription factor responsible for specification of satellite cells to the myogenic lineage) was upregulated after myotoxin injection, with the increase in expression peaking at 5 days post-injury. Furthermore, expression of Myf5 was significantly increased in the regenerating muscles of β_1_/β_2_-KO mice than controls, indicating that myoblast proliferation was not compromised in the β_1_/β_2_-KO mice, and may be propagated for longer after muscle injury, than in control mice. The induction of MyoD (which initiates the terminal differentiation program in myoblasts) and myogenin (which is involved in the activation of muscle-specific genes in the immature muscle cells [37]) following injury was also exaggerated in the muscles of β_1_/β_2_ double-KO mice. Taken together, our MRF expression data suggest that myoblast proliferation and differentiation may be enhanced in β_1_/β_2_-KO mice at the expense of moderately delayed differentiation. This observation, consistent with what we had expected and described in a previous review[38], may explain why force producing capacity is impaired at 7 days post-injury in β_1_/β_2_-KO mice, but that muscles are capable of restoring functionality (relative to uninjured muscles) similar to control animals at 10 days post-injury. This rapid ‘catch up’ where the muscles of β_1_/β_2_-KO mice seemingly overcome their initial delayed regeneration and function impairment, is supported by our observations of cultured primary myoblasts from β_1_/β_2_-KO mice, where proliferation was enhanced and prolonged and differentiation was delayed.

The present study utilized whole body β_1_/β_2_-KO mice, since to our knowledge there are no muscle-specific β_1_/β_2_-KO mice currently available. One concern with using the whole body β_1_/β_2_-KO mouse (a concern that is also valid in any in vivo study of β-agonist administration) is that any effects on muscle regeneration may be a consequence of perturbations of non-muscle physiological systems, rather than a direct effect on muscle regeneration *per se*. For example, the altered inflammatory response observed in the present study, while not a direct result of the muscle lacking β-ARs, undoubtedly influenced fiber regeneration.

To obviate these concerns we isolated myoblasts from both β_1_/β_2_-KO mice and C57BL/6 controls to examine myoblast proliferation and differentiation in the absence of confounding factors, and found that myoblasts isolated from β_1_/β_2_-KO mice proliferated more rapidly and differentiated far less effectively than those from C57BL/6 controls. While initially this may seem to be at odds with our MRF expression data from regenerating muscles, it must be remembered that even if myoblast differentiation was impaired in vivo, the vastly greater number of myoblasts present in the muscle due to the increased proliferation would still result in an overall increase in MyoD and myogenin expression in the muscle. Interestingly, we have previously documented a dramatic increase in the gene expression of adrb1 and adrb2 (β_1_- and β_2_-adrenoceptors, respectively) during the switch from proliferation to differentiation [38]. Combined with the findings of the present study, these data support a role for β_1_/β_2_-ARs in inhibiting myoblast proliferation and promoting differentiation.

Another concern with whole body β_1_/β_2_-KO mice is the potential for cardiovascular disturbances (particularly alterations in local blood flow) to influence muscle regeneration. We do not believe that the muscles from β_1_/β_2_-KO mice suffered a significant deficit in perfusion as this would have resulted in a constant inhibition of regeneration, whereas we observed a deficit in regeneration only at 7 days post-injury. In fact, the muscles from β_1_/β_2_-KO mice subsequently regenerated faster than control in order to ‘catch up’ to the control muscles at the later time points.

With all our data taken together we propose a model in which β_1_/β_2_-KO mice exhibit an enhanced inflammatory response to injury and enhanced myoblast proliferation during regeneration. We believe that β_1_/β_2_-KO mice have delayed early regeneration due to the prolonged and enhanced inflammatory response, but once the inflammation subsides, the enhanced myoblast proliferation allows the β_1_/β_2_-KO muscles to regenerate rapidly – thus accounting for the rapid ‘catch up’ of muscles between 7 and 10 days post-injury. Further studies using muscle specific knockdown of β-ARs or virally-mediated ‘knock in’ of β-ARs to inflammatory cells of β_1_/β_2_-KO mice, or other injury models (such as crush or burn injuries) with a higher inflammatory component would help test this hypothesis.

In summary, our findings indicate that β-ARs play an important role in early muscle regeneration, at least in part via a direct effect on myoblast proliferation and differentiation. Manipulation of β-AR signaling during these early stages of regeneration may therefore improve the rate, extent and efficacy of the regenerative process, to enhance functional recovery after injury.

## Supporting Information

Figure S1 **Maximal tetanic force of regenerating TA muscles of different strains of β-KO mice following injury and expressed as absolute force (P_o_).** Force production by β_1_-KO mice did not differ from controls during regeneration (A), but both β_2_-KO mice (B) and β_1_/β_2_-KO mice (C) had significant force deficits during all stages of regeneration (#*P*<0.05, strain main effect, 2-way ANOVA).
(TIF)

Figure S2 **Frequency-force relationships for different β-KO mouse strains.** β_1_-KO mice did not have altered frequency-force relationship compared with controls (A and B), but β_2_-KO (C and D) and β_1_/β_2_-KO mice (E and F) produced significantly lower forces at all frequencies when compared with controls. (*^#^P*<0.05, strain main effect, 2-way ANOVA).
(TIF)
