# Supplementary figures and images for: Functional β-Adrenoceptors Are Important for Early Muscle Regeneration in Mice through Effects on Myoblast Proliferation and Differentiation
Source: PLoS One. 2014 Jul 7;9(7):e101379. doi: 10.1371/journal.pone.0101379 (PMC4084885; doi:10.1371/journal.pone.0101379)

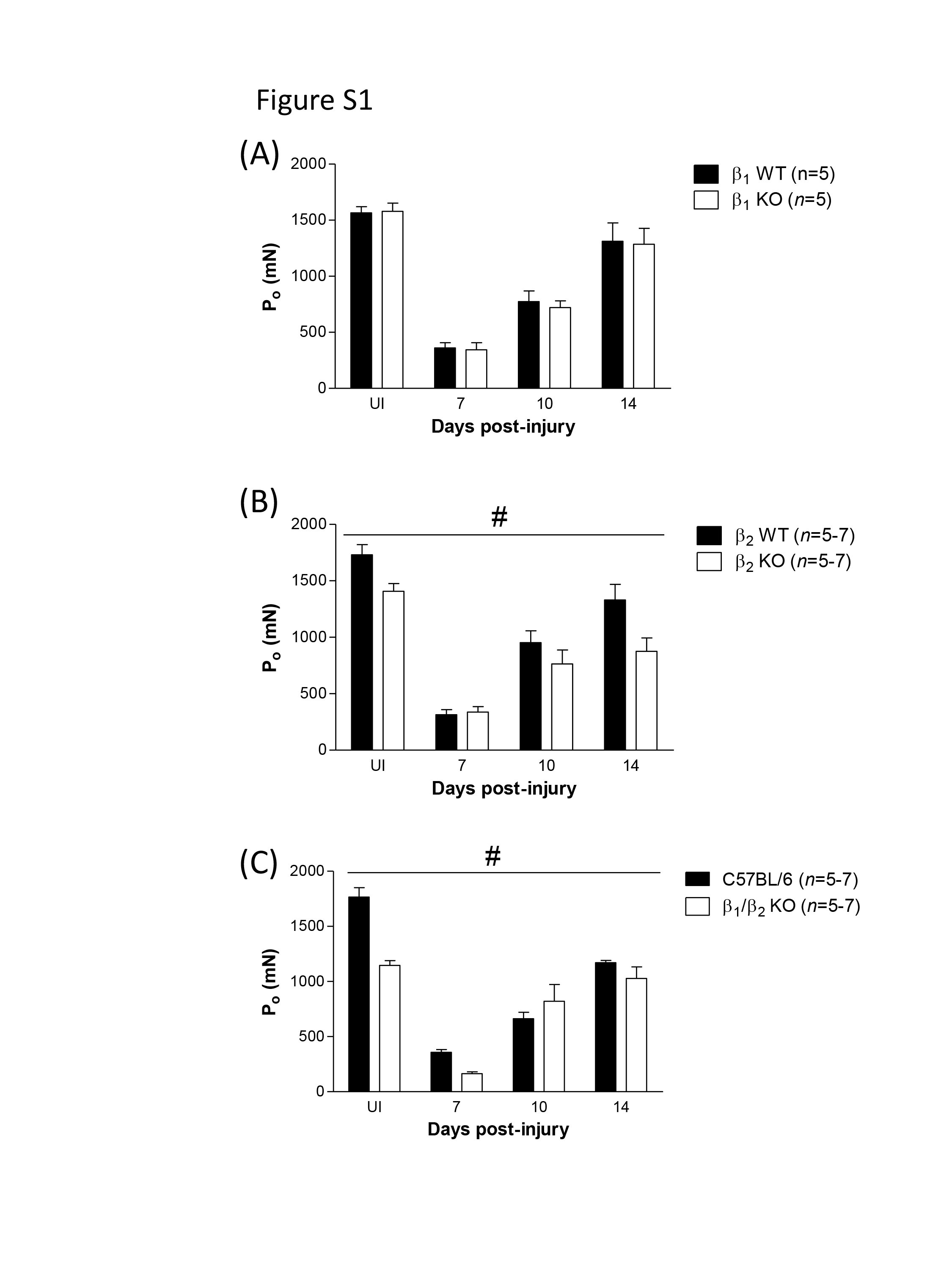

Supplement: Figure S1 — Maximal tetanic force of regenerating TA muscles of different strains of β-KO mice following injury and expressed as absolute force (Po). Force production by β1-KO mice did not differ from controls during regeneration (A), but both β2-KO mice (B) and β1/β2-KO mice (C) had significant force deficits during all stages of regeneration (#P<0.05, strain main effect, 2-way ANOVA). (TIF) [file pone.0101379.s001.tif]

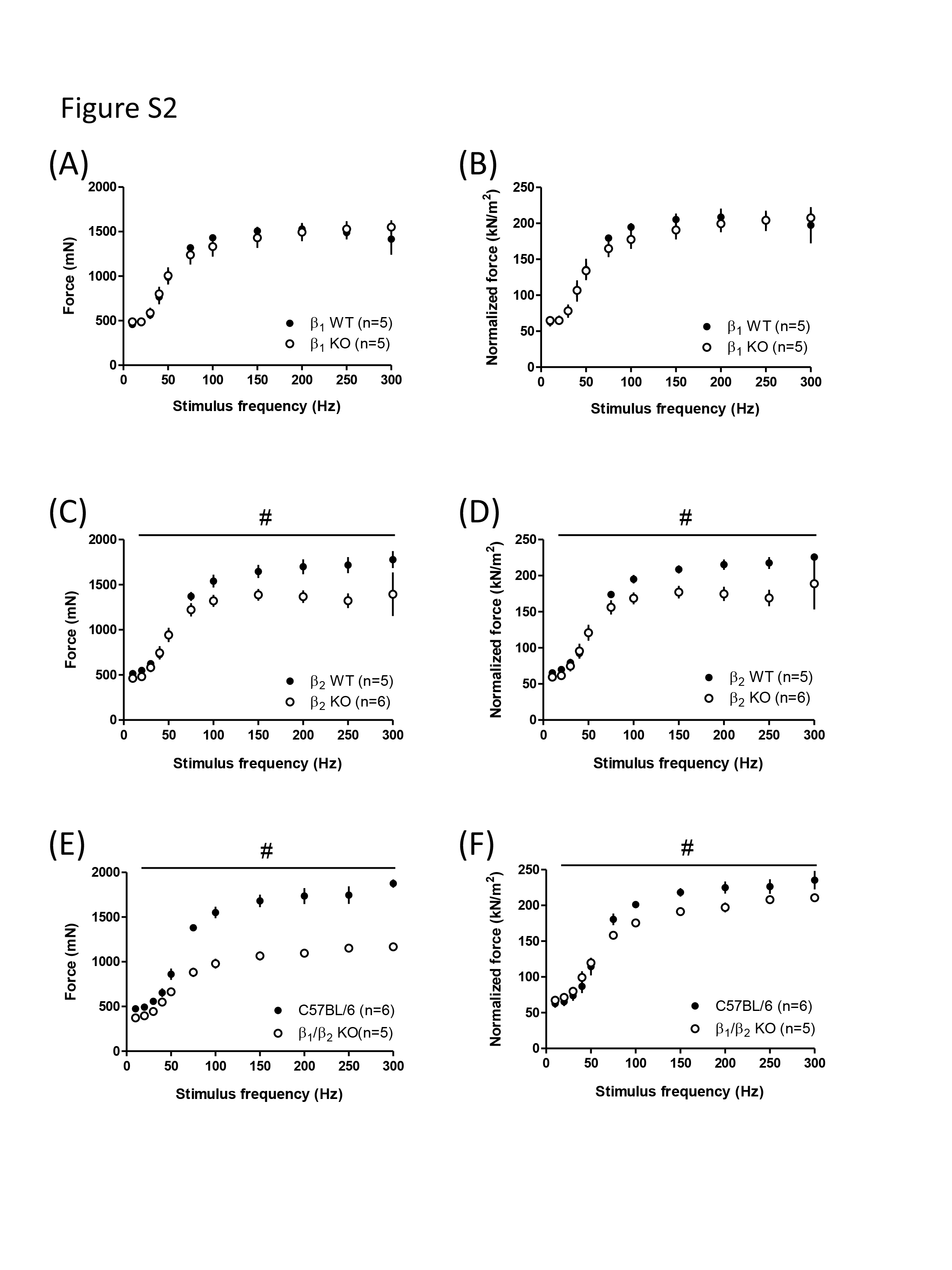

Supplement: Figure S2 — Frequency-force relationships for different β-KO mouse strains. β1-KO mice did not have altered frequency-force relationship compared with controls (A and B), but β2-KO (C and D) and β1/β2-KO mice (E and F) produced significantly lower forces at all frequencies when compared with controls. (#P<0.05, strain main effect, 2-way ANOVA). (TIF) [file pone.0101379.s002.tif]
